# Golgi Phosphoprotein 3 Mediates Radiation-Induced Bystander Effect via ERK/EGR1/TNF-α Signal Axis

**DOI:** 10.3390/antiox11112172

**Published:** 2022-11-01

**Authors:** Feng Qin, Guodong Chen, Kwan Ngok Yu, Miaomiao Yang, Wei Cao, Peizhong Kong, Shengjie Peng, Mingyu Sun, Lili Nie, Wei Han

**Affiliations:** 1 Anhui Province Key Laboratory of Medical Physics and Technology, Institute of Health and Medical Technology, Hefei Institutes of Physical Science, Chinese Academy of Sciences, Hefei 230031, China; 2 Scinece Island Branch, Graduate School of USTC, Hefei 230026, China; 3 Institute of Sericultural, Anhui Academy of Agricultural Sciences, Hefei 230061, China; 4 Hefei Cancer Hospital, Chinese Academy of Sciences, Hefei 230031, China; 5 Department of Physics, City University of Hong Kong, Tat Chee Avenue, Kowloon Tong 999077, Hong Kong; 6 State Key Laboratory in Marine Pollution, City University of Hong Kong, Tat Chee Avenue, Kowloon Tong 999077, Hong Kong; 7 Collaborative Innovation Center of Radiation Medicine of Jiangsu Higher Education Institutions and School for Radiological and Interdisciplinary Sciences (RAD-X), Soochow University, Suzhou 215006, China

**Keywords:** GOLPH3, radiation-induced bystander effect, TNF-α, EGR1

## Abstract

The radiation-induced bystander effect (RIBE), an important non-targeted effect of radiation, has been proposed to be associated with irradiation-caused secondary cancers and reproductive damage beyond the irradiation-treated area after radiotherapy. However, the mechanisms for RIBE signal(s) regulation and transduction are not well understood. In the present work, we found that a Golgi protein, GOLPH3, was involved in RIBE transduction. Knocking down GOLPH3 in irradiated cells blocked the generation of the RIBE, whereas re-expression of GOLPH3 in knockdown cells rescued the RIBE. Furthermore, TNF-α was identified as an important intercellular signal molecule in the GOLPH3-mediated RIBE. A novel signal axis, GOLPH3/ERK/EGR1, was discovered to modulate the transcription of TNF-α and determine the level of released TNF-α. Our findings provide new insights into the molecular mechanism of the RIBE and a potential target for RIBE modulation.

## 1. Introduction 

Malignant tumors are the main cause of death in the world, and radiotherapy is widely used in tumor treatment. In radiotherapy, DNA damages are induced by ionizing radiation (IR) in the targeted cells, including cancer cells and the surrounding normal cells, which eliminate the tumor and/or cause normal tissue injury. Except for the targeted effects, some biological events, including genomic instability, epigenetic changes, DNA methylation, tumor formation, etc. [1], in the non-irradiated cells/tissues were observed beyond the irradiated area. These effects in non-irradiated cells or tissues, termed the radiation-induced bystander effect (RIBE), implies that the radiation-induced biological effect on cells or tissues is not limited to the targeted cells. It is becoming clear that the RIBE could be triggered by low- or high-dose radiation and occurs not only in adjacent tissues but also in distant tissues [2]. Furthermore, mutations and genomic instability in the bystander cells tend to increase the risk of carcinogenesis [3]. Epidemiological studies indicate that approximately 1 in 70 patients surviving more than 10 years after prostate cancer radiotherapy will develop secondary cancer. In addition to the tissues adjacent to the radiation field, secondary tumor formation is also found in the tissues distant from the radiation field. Conceptually, RIBE contributes to radiation-induced secondary carcinogenesis [4]. Mancuso et al. [3] further reported that DNA double-strand breaks (DSBs) and apoptotic cell death in the non-irradiated cerebellum were caused by irradiation, with the skull shielded, in Patched-1+/− (Ptch1+/−) mice, thereby resulting in a significant increase in the medulloblastoma rate (39%) compared to the sham-treated group. The study provides direct evidence for the relationship of cancer induction and the RIBE. Recently, the damages in distant sperm cells triggered by the RIBE result in significant epigenetic changes in offspring without IR treatment [5], suggesting that the RIBE may also be a potential hazard to the reproductive system. Zhang et al. [6] found that the number and viability of the sperm in male mice was affected by the irradiation distant from the testes, causing a decrease in the average number of offspring. With the significant prolongation of the disease-free period of cancer patients, the potential health risks induced by the RIBE raise concerns after radiotherapy, so developing strategies to modulate the RIBE will be indispensable. 

Extensive studies in vitro and in vivo have investigated the possible mechanisms of the RIBE. In the presence of direct cell-to-cell contact, the gap junction provides communication channels for the RIBE signals transmitting from irradiated to non-irradiated cells [7]. Additionally, soluble factors released from irradiated cells [8,9,10,11,12], transforming growth factor-β (TGF-β), tumor necrosis factor-α (TNF-α), interleukin-6 (IL-6), interleukin-8 (IL-8), microRNAs, nitric oxide (NO), cysteine protease CPR-4, etc., also mediate RIBE by activating some specific signaling pathways, including NF-κB, MAPK, STAT3, etc., in the bystander cells. Moreover, increasing evidence also suggests that the production of the soluble factors involved in the RIBE are regulated by various pathways in the processes of oxidative stress, energy metabolism, membrane vesicle transport, etc., in irradiated cells. In these signal pathways, some important molecules, such as SirT1 [13], p53 [14] and HIF-1α [15], in irradiated cells have been revealed as potential targets to modulate the RIBE. Although the RIBE has been extensively studied, further in-depth investigations on its underlying mechanisms are needed to understand it well. 

Golgi phosphoprotein 3 (GOLPH3), also known as MIDAS, GPP34 or GMx33, is highly located at the trans face of the Golgi cisternae and exerts diverse biological functions, such as Golgi morphology maintenance, protein trafficking, receptor recycling and glycosylation. Recently, GOLPH3 has been recognized as a new proto-oncogene, and overexpression of GOLPH3 has been observed in a variety of malignant tumors including ovarian cancer, breast cancer, prostate cancer, liver cancer, etc. [16,17]. The high expression of GOLPH3 not only enhances the proliferation and the invasion of cancer cells [18] but also promotes radiochemotherapy resistance [19], thus leading to cancer progression and poor prognosis. All these studies indicate that GOLPH3 can serve as a potential target in clinical tumor therapy. In addition, GOLPH3 has been reported to be elevated during oxidative stress and play an important role in Golgi apparatus (GA) stress [20]. In an oxygenglucose deprivation/reoxygenation (OGD/R) model, GOLPH3 functions as a sensor of GA stress and further boosts the ROS production, thereby triggering apoptosis [21]. These findings indicate that GOLPH3 is also a regulator of oxidative stress. Importantly, GOLPH3 has also been proposed to be involved in the regulation of secreted factors. For instance, knockdown of GOLPH3 decreases the metastasis-related factors, matrix metalloproteinase-2 (MMP-2), matrix metalloproteinase-9 (MMP-9) and matrix metalloproteinase-14 (MMP14), at the mRNA level or protein level [22,23]. GOLPH3 mediates the release of PI4KIIIβ-dependent secreted factors (CLU, PLOD3, TIMP1, SEMA3C and STC2), which may act as pro-survival and pro-metastatic effectors [24]. In addition, GOLPH3 also modulates the expression of miRNAs in exosomes and promotes exosomal-WNT3A secretion [25,26]. Thus, GOLPH3 is believed to have the potential to change the tumor microenvironment.

In this study, we investigated the role of GOLPH3 in the RIBE. Knocking down GOLPH3 in the irradiated cells significantly attenuated the RIBE in the bystander cells, whereas the re-expression of GOLPH3 in GOLPH3 knockdown cells restored the RIBE, suggesting the important role of GOLPH3 in the RIBE. Mechanistically, we discovered that GOLPH3 regulated the expression and release of TNF-α via the ERK/EGR1 pathway to act as an extracellular signal to mediate the RIBE. We hope our research can provide new insights to understand the underlying mechanism of RIBE and give some hints to protect normal tissues in radiotherapy.

## 2. Materials and Methods

### 2.1. Cell Culture

The human lung cancer cell lines (A549 and NCI-H1299) were purchased from the Cell Bank of Type Culture Collection of the Chinese Academy of Sciences (Shanghai, China), cultured in RPMI 1640 medium (Gibco, Carlsbad, CA, USA, Cat#C11875500BT) and supplemented with 10% fetal bovine serum (FBS, Lonsera, Uruguay, Cat#S711-001S), 100 μg/mL streptomycin and 100 U/mL penicillin (Gibco, Cat#15140-122). Human lung fibroblasts (NHLF) cells were cultured in DMEM medium (Gibco, Cat#12100046) supplemented with 10% FBS, 100 μg/mL streptomycin and 100 U/mL penicillin. All cells were maintained at 37 °C incubator with humidified atmosphere of 5% CO2. All cell lines have been examined for mycoplasma contamination.

### 2.2. Irradiation 

The cells were irradiated with a XHA600D X-ray irradiator (SHINVA, Zibo, Shandong, China) at a dose rate of 0.189 Gy/min. Before irradiation, the culture medium was replaced with the fresh medium. A dose of 10 Gy was used in all subsequent experiments. 

### 2.3. Model of RIBE In Vitro

**Figure 1:**
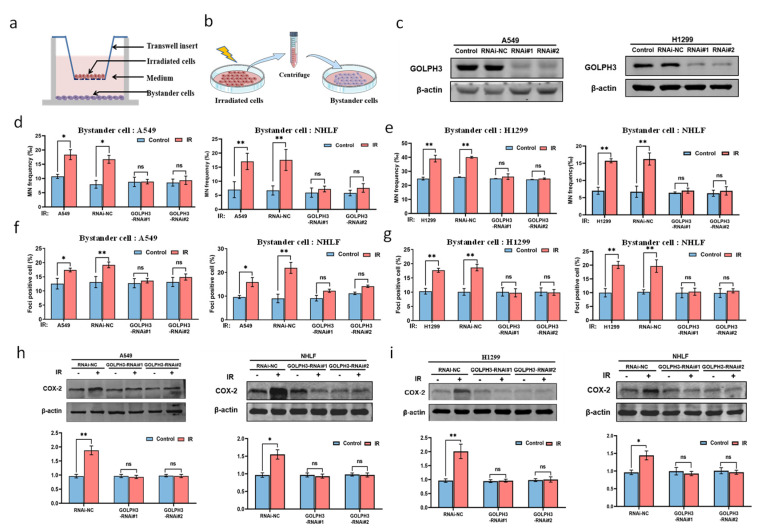
GOLPH3 in the irradiated cells mediates the occurrence of RIBE. (**a**) Transwell co-culture system; (**b**) model of conditioned medium transfer; (**c**) knockdown of GOLPH3 validated by Western blotting in A549 and H1299 cells; (**d**) MN yields in the bystander cells (A549 or NHLF) after co-culture with the irradiated A549, A549/RNAi-NC or A549/GOLPH3-RNAi cells, respectively; (**e**) MN yields in the bystander cells (H1299 or NHLF) after co-culture with the irradiated H1299, H1299/RNAi-NC and H1299/GOLPH3-RNAi cells, respectively; (**f**) quantification of γ-H2AX positive cells in the bystander cell population (A549 or NHLF) after 3 h co-culture with the irradiated A549, A549/ RNAi-NC and A549/GOLPH3-RNAi cells, respectively; (**g**) quantification of γ-H2AX-positive cells in the bystander cells (H1299 or NHLF) after 3 h co-culture with the irradiated H1299, H1299/RNAi-NC and H1299/GOLPH3-RNAi cells, respectively; (**h**) representative results of Western blot (upper) and the corresponding quantification (lower) of COX-2 protein in the bystander cells (A549 or NHLF) after 24 h co-culture with irradiated A549/RNAi-NC and A549/GOLPH3-RNAi cells, respectively; (**i**) representative result of Western blot (upper) and the corresponding quantification (lower) of COX-2 protein in the bystander cells (H1299 or NHLF) after 24 h co-culture with the irradiated H1299/RNAi-NC and H1299/GOLPH3-RNAi cells, respectively. *: *p* < 0.05; **: *p* < 0.01; ns: not significant.

In the Transwell co-culture system, cells were seeded into the Transwell insert (2 × 10^5^ cells/insert) and the corresponding 6-well plate (5 × 10^4^ cells/well), respectively. After overnight incubation, the culture medium was replaced with fresh medium. Subsequently, the cells in the Transwell insert were irradiated with X-rays, immediately followed by co-culture with the cells (bystander) in the plate (Figure 1a).

In the medium transfer experiment, the conditioned medium was collected from the irradiated cells at the indicated time points, followed by centrifugation at 3000 g for 5 min. The supernatants were collected and then transferred to treat the bystander cells (Figure 1b). 

### 2.4. Establishment of Stable Cell Lines

The plasmids, GV248-NC (the negative control), GV248-hGOLPH3-RNAi#1 and GV248-hGOLPH3-RNAi#2, were constructed to knockdown human GOLPH3 (hGOLPH3), as described in our previous study [27]. The shRNA target sequences of hGOLPH3 were as follows: GCATGTTAAGGAAACTCAGCC (RNAi#1) and GCAGCGCCTCATCAAGAAAGT (RNAi#2). Lentiviruses were produced in HEK 293T cells by co-transfecting GOLPH3 shRNA constructs, which contained shRNAs against hGOLPH3 or mGOLPH3, together with the packaging plasmids (psPAX2 and pMD2.G). Cells (A549 and H1299) were infected with the lentiviruses, and the stable cell lines expressing GOLPH3 shRNA were established after selection with puromycin. 

### 2.5. Rescue Experiment

The recombinant adenovirus expression vector of GOLPH3 (pHBAd-mCMV-3×Flag-hGOLPH3Res-IRES-EGFP), containing a GOLPH3 isoform with silent mutations in the GOLPH3-RNAi#2-binding sites (hGOLPH3-Res), was also constructed for adenovirus package in our previous study [27]. The adenovirus expressing the hGOLPH3-Res gene was prepared by Hanbio Co., Ltd. (Shanghai, China). After adenovirus infection to rescue the expression of GOLPH3, RIBE was detected in the stable GOLPH3 knockdown cell lines (hGOLPH3-RNAi#2). 

### 2.6. Knockout of GOLPH3 by Using CRISPR/Cas9 

The sgRNA (5-CACCGCCGCTACCGTGAGTTCGTG-3) targeted to GOLPH3 was cloned into lentiCRISPRv2 vector (Addgene, MA, USA, Cat#52961) and validated sequencing. The constructed sgRNA lentiviral vector and the packaging plasmids (psPAX2 and pMD2.G) were co-transfected into HEK293T cells to produce lentiviruses. The lentiviruses were used to infect A549 cells, followed by puromycin selection (2 μg/mL) for 7 days. After single-cell culture, single-cell clones were obtained and subjected to sequencing for the target sequence. Finally, the GOLPH3 knockout cell line (A549/GOLPH3-KO) was generated and then utilized for further experiments.

### 2.7. Western Blotting 

The whole cell protein was extracted with RIPA lysis buffer (Beyotime Biotechnology, Shanghai, China, Cat#S711-001S), and the protein concentration was measured with BCA protein analysis kit (Beyotime Biotechnology, Cat#P0012). Equal amounts of total protein were loaded on 6%–15% sodium dodecyl sulfate-polyacrylamide gel electrophoresis (SDS-PAGE), and then the separated proteins were transferred to PVDF membrane (Merck Millipore, Darmstadt, Germany, Cat#IPVH00010). After blocking with 5% skim milk, membranes were incubated with primary antibodies at 4 °C overnight, followed by incubation with the IRDye conjugated secondary antibody for 1 h at room temperature. The antibodies used in this study were as follows: Anti-β-Actin (1:1000, Abcam, Cambridge, MA, USA, Cat#ab97626), Anti-GOLPH3 (1:1000, Abcam, Cat#ab91492), Anti-COX-2 (1:1000, Proteintech, WuHan, China, Cat#27308-1-AP), Anti-iNOS (1:1000, Proteintech, Cat#18985-1-AP) and IRDye-conjugated (680RD Goat anti-Mouse/800CW Goat anti-Rabbit) IgG secondary antibodies (1:10000, Li-COR Biosciences, Lincoln, NE, USA, Cat#926-68070/Cat#926-32211). Membranes were imaged with an Odyssey CLX Infrared Imaging System (Li-COR Biosciences).

### 2.8. ELISA Assay

Concentrations of human TGF-β (Elabscience, Wuhan, China, Cat#E-EL-0162c), TNF-α (Elabscience, Cat#E-EL-H0109c) and IL-6 (Elabscience, Cat#E-EL-H6156) secreted from the irradiated cells were measured with ELISA kits, in accordance with the instructions of the manufacturer. Briefly, cell culture media were collected and centrifuged at 1000 g (20 min, 4 °C) to remove cell debris. The supernatants were collected for ELISA assays.

### 2.9. Micronucleus (MN) Test

The frequency of micronucleus formation was assessed with the cytokinesis-block micronucleus technique [28]. Briefly, 5 × 10^4^ bystander cells were seeded into each well of 6-well plate. After co-culture with irradiated cell, cytochalasin B (Sigma, Saint Louis, Missouri, USA, Cat#C6762) was added into the culture medium with a final concentration of 1 μg/mL, and then the cells were fixed with 4% paraformaldehyde (Sigma, Cat#P6148-100G) 48 h later (two doubling times) and stained with 0.1% acridine orange (Sigma, Cat#A6014) solution. Finally, the cells were viewed under a DMI4000B fluorescence microscope (Leica, Wetzlar, Germany). The micronuclei in at least 1000 bi-nucleated (BN) cells were counted, and the frequencies of MN per 1000 BN cells were calculated.

### 2.10. Quantitative Real-Time PCR (qPCR)

Total RNA was extracted with a Biospin SimplyP Total RNA Extraction Kit (Bioer Technology, HangZhou, China, Cat#BSC63S1), in accordance with the instructions of the manufacturer. Sequentially, the concentration and purity of total RNA were analyzed with a NanoDrop 2000 (Thermo Fisher Scientific, Waltham, MA, USA). The total RNA (2 μg) was used for first strand cDNA synthesis using Hifair II 1st Strand cDNA Synthesis SuperMix (YEASEN, ShangHai, China, Cat#11120ES60). The qPCR was performed with the Hieff qPCR SYBR Green Master Mix (YEASEN, Cat#11201ES) on a Roche 480 Light Cycler (Roche, Basel, Switzerland). The specified primers sequences were as follows: 5′-TGTAAGTCAGATGCTCCAACAGG-3′, 5′-TCACCCATTTGTCAAGAACGG-3′ (GOLPH3), 5′-CCTCTCTCTAATCAGCCCTCTG-3′, 5′-GAGGACCTGGGAGTAGATGAG-3′ (TNF-α), 5′-CTGGGACGACATGGAGAAAA-3′, 5′-AAGGAAGGCTGGAAGAGTGC-3′ (β-actin).

### 2.11. Immunofluorescence 

After being co-cultured with the radiated cells, the bystander cells were fixed with 4% paraformaldehyde for 30 min and permeabilized with TNBS solution (PBS added with 0.5% Triton X-100 and 1% FBS) for 1 h. Subsequently, the cells were incubated with anti-γ-H2AX antibodies (1:1000, Abcam, Cat#ab81299) at 37 °C for 1 h. After washing with TNBS, the cells were incubated with a fluorescent secondary antibody: Goat anti rabbit IgG H&L (TRITC) (1:1000, Abcam, Cat#ab6718) at 28 °C for 1 h, followed by incubation with DAPI (5 mg/mL, Sigma, Cat#D9542) for 15 min. Images were captured with a fluorescence microscope (DMI4000B, Leica). At least 500 cells per slide were counted.

### 2.12. Statistical Analysis

All experiments were repeated independently at least 3 times. The results were expressed as mean ± standard deviation. The difference was calculated with Student’s *t*-test or one-way ANOVA using SPSS v21.0 software (SPSS, USA). *p* < 0.05 indicates a statistically significant difference. 

## 3. Results

### 3.1. GOLPH3 Plays a Key Role in the Production of RIBE Signals Released from Irradiated Cells 

To explore the relationship between GOLPH3 and RIBE in vitro, we stably knocked down GOLPH3 expression in A549 and H1299 cells with two independent short hairpin RNAs (GOLPH3-RNAi#1 and GOLPH3-RNAi#2), validated by Western blot (Figure 1c). The RNAi-NC and GOLPH3-RNAi cells were irradiated and cocultured with the bystander cells. The bystander A549 or H1299 cells were used to determine the RIBE in cancer cells, and the bystander NHLF cells were used to determine the RIBE in normal lung cells. The frequency of MN and DSBs (γ-H2AX foci positive cells) was then used to assess the RIBE. The results in Figure 1d show that the increase in MN yields was observed in the bystander cells (A549 or NHLF) after co-culture with the irradiated GOLPH3 wild-type cells (A549 and A549/RNAi-NC), whereas knockdown of GOLPH3 in irradiated cells (A549/GOLPH3-RNAi) significantly attenuated the formation of MN in the bystander cells. A similar phenomenon was also observed in the bystander cells (H1299 or NHLF) co-cultured with GOLPH3 wild-type or knockdown H1299 cells (Figure 1e). In addition, the fraction of γ-H2AX positive cells also increased in the bystander cells (A549, H1299 or NHLF), which were co-cultured with irradiated GOLPH3 wild-type cells, whereas the fraction of γ-H2AX-positive cells was dramatically reduced in the bystander cells co-cultured with irradiated GOLPH3 knockdown cells (Figure 1f,g). These results indicate that knocking down GOLPH3 in the irradiated cells significantly blocks the occurrence of the RIBE. This was also supported by the results that COX-2 protein, an important mediator of the RIBE, was upregulated in the bystander cells by the RIBE signals released from irradiated GOLPH3 wild-type cells, but the induction of COX-2 was reversed after knockdown of GOLPH3 in the irradiated cells (Figure 1h,i).

**Figure 2:**
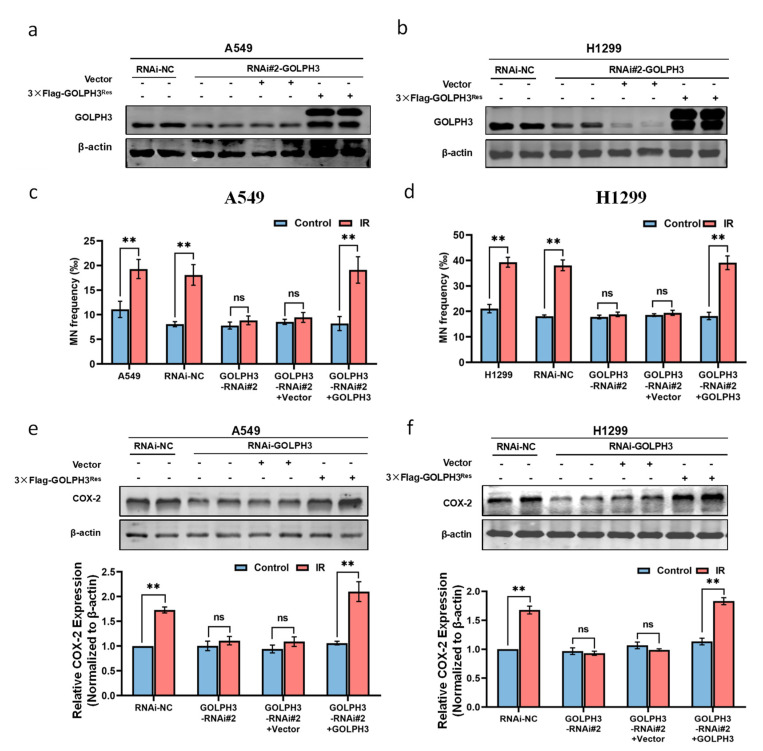
Re-expression of GOLPH3 in the irradiated GOLPH3 knockdown cells rescues RIBE. (**a**,**b**) Re-expression of GOLPH3 protein in GOLPH3 knockdown cell lines (A549/GOLPH3-RNAi#2 and H1299/GOLPH3-RNAi#2); (**c**) MN yields in the bystander A549 cells after co-culture with the irradiated A549/GOLPH3-RNAi#2 cells with GOLPH3 re-expression; (**d**) MN yields in bystander H1299 cells after co-culture with the irradiated H1299/GOLPH3-RNAi#2 cells with GOLPH3 re-expression; (**e**) representative Western blot and corresponding quantification of COX-2 protein in the bystander A549 cells after co-culture with the irradiated GOLPH3-RNAi#2 cells with GOLPH3 re-expression; (**f**) representative Western blot and corresponding quantification of COX-2 protein in the bystander H1299 cells after co-culture with the irradiated GOLPH3-RNAi#2 cells with GOLPH3 re-expression. **: *p* < 0.01; ns: not significant.

To further confirm the key role of GOLPH3 in RIBE, the rescue experiment was performed by re-expressing exogenous GOLPH3Res-3×Flag in GOLPH3 knockdown cells (Figure 2a,b). Our results showed that the MN yields in the bystander cells were restored after re-expression of GOLPH3 in the irradiated GOLPH3 knockdown cells (Figure 2c,d). Moreover, re-expression of GOLPH3 in the irradiated A549/GOLPH3-RNAi#2 or H1299/GOLPH3-RNAi#2 cells significantly elevated the expression of COX-2 in the bystander cells (Figure 2e,f). These results indicate that the re-expression of GOLPH3 in the irradiated GOLPH3 knockdown cells effectively rescues the RIBE, confirming that GOLPH3 is crucial to the production of the RIBE signals in the irradiated cells.

### 3.2. TNF-α Acts as an Intercellular Molecule in GOLPH3-Mediated RIBE

**Figure 3:**
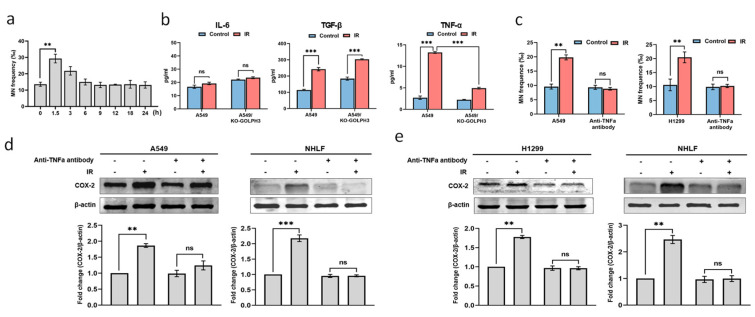
GOLPH3 modulates RIBE via TNF-α released from the irradiated cells. (**a**) MN yields in A549 cells after treatment with CM harvested at indicated timepoints post radiation; (**b**) ELISA detection of TNF-α, TGF-β and IL-6 in 1.5 h CM harvested from the irradiated A549 or A549/GOLPH3-KO cells; (**c**) MN yields in the bystander cells (A549 or H1299) treated by 1.5 h CM harvested from the irradiated A549 or H1299 cells with or without anti-TNF-α antibody supplement; (**d**) representative Western blot and corresponding quantification of COX-2 in the bystander A549 or NHLF cells treated with 1.5 h CM harvested from irradiated A549 cells with or without anti-TNF-α antibody supplement; (**e**) representative Western blot and corresponding quantification of COX-2 in bystander H1299 or NHLF cells treated with 1.5 h CM harvested from the irradiated H1299 cells with or without anti-TNF-α antibody supplement. **: *p* < 0.01; ***: *p* < 0.001; ns: not significant.

To explore the nature of signal molecule(s) released by the irradiated cells, we transferred the conditioned medium (CM) at the indicated time points after IR to treat the bystander cells. As shown in Figure 3a, the CM harvested at 1.5 h after IR elevated MN frequency to a peak level in the bystander cells. It is well-known that RIBE could be mediated by the soluble factors (such as TNF-α, TGF-β, IL-6, etc.) released by irradiated cells [8,9,10,11,12]. Hence, a GOLPH3 knockout cell line (A549/KO-GOLPH3) was established (Supplementary Figure S1A,B), and then the TNF-α, TGF-β and IL-6 in the 1.5 h CM harvested from the irradiated A549 or A549/KO-GOLPH3 were measured with ELISA. Interestingly, a significant increase in TNF-α and TGF-β rather than IL-6 was observed in the 1.5 h CM harvested from the irradiated A549 cells, and knocking out GOLPH3 decreased the amount of TNF-α significantly (Figure 3b). These results suggest that GOLPH3 regulates the release of TNF-α from irradiated cells. To further verify the role of TNF-α in the transduction of the GOLPH3-mediated RIBE, a neutralizing antibody against TNF-α was added into the 1.5 h CM harvested from GOLPH3 wild-type cells (A549 or H1299) before treating the bystander cells. The addition of the neutralizing antibody against TNF-α significantly suppressed the formation of MN (Figure 3c) in the bystander cells, demonstrating that blocking TNF-α inhibited the transduction of the RIBE. This was also supported by the results that the addition of the neutralizing antibody against TNF-α also significantly attenuated the COX-2 induction in the bystander cells treated by the 1.5 h CM harvested from irradiated the A549 or H1299 cells (Figure 3d,e). These results indicate that the TNF-α released from irradiated cells may act as an intercellular signal molecule to mediate the RIBE transduction.

### 3.3. GOLPH3 Regulates the Transcription of TNF-α via ERK/EGR1 Pathway

**Figure 4:**
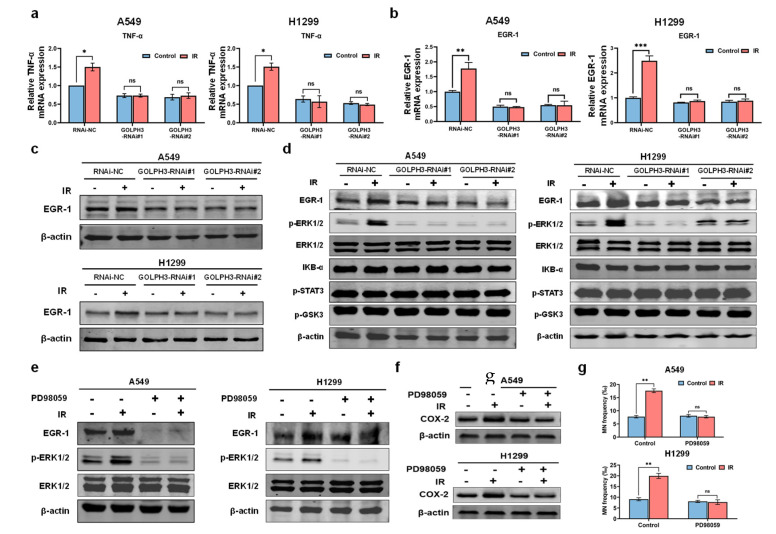
GOLPH3 regulates the transcription of TNF-α via ERK/EGR1 pathway. (**a**,**b**) Relative mRNA expression of TNF-α (**a**) and EGR1 (**b**) after IR in RNAi-NC, GOLPH3-RNAi#1 and GOLPH3-RNAi#2 cells, respectively; (**c**) the expression of EGR1 protein after irradiation; (**d**) detection of ERK, STAT3, GSK3β and NF-κB pathway activation in the indicated cells (1.5 h after IR); (**e**) EGR1 expression in irradiated A549 and H1299 cells with or without the ERK inhibitor (PD98059) treatment; (**f**) COX-2 expression in the bystander cells (A549 or H1299) after treating the irradiated cells with the ERK inhibitor; (**g**) MN yields in the bystander cells (A549 or H1299) after treating the irradiated cells with the ERK inhibitor. *: *p* < 0.05; **: *p* < 0.01; ***: *p* < 0.001; ns: not significant.

**Figure 5:**
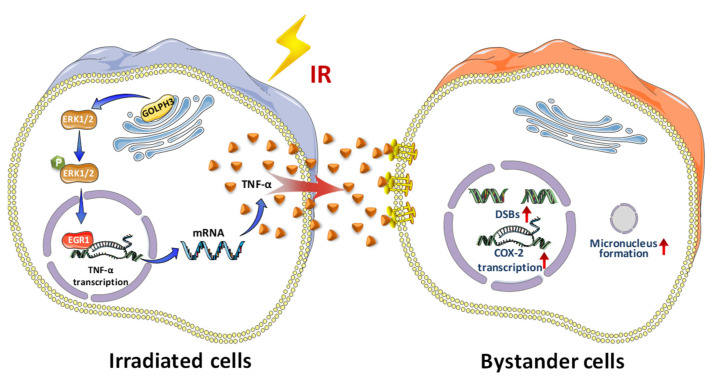
Schematic diagram of GOLPH3-mediated RIBE. In the irradiated cells, GOLPH3 modulates the activation of ERK1/EGR1 pathway to promote the transcription of TNF-α; the secreted TNF-α from the irradiated cell acts as an intercellular signal molecule to mediate the induction of RIBE.

To determine how GOLPH3 regulates TNF-α, we firstly detected the IR-induced transcriptional activation of TNF-α after knocking down GOLPH3. Intriguingly, our results showed that IR enhanced the transcription of TNF-α, but loss of GOLPH3 significantly abolished it after IR (Figure 4a). Given that Egr-1, an “immediate-early response” protein after IR treatment, is a critical transcription factor of TNF-α, the expression of EGR1, at both the mRNA and protein levels, was detected after IR in RNAi-NC and RNAi-GOLPH3 cells. As expected, knocking down GOLPH3 disabled upregulation of the protein and mRNA levels of EGR1 after IR (Figure 4b,c), suggesting the key role of GOLPH3 in EGR1 activation. To explore the possible mechanism of GOLPH3 modulating EGR1, we screened the activation of several pathways previously reported to be associated with GOLPH3. As shown in Figure 4d, ERK1/2 (but not GSK3β, STAT3 or IκB) was activated after IR, and GOLPH3 knockdown significantly blocked the activation of ERK1/2. Furthermore, a selective ERK1/2 inhibitor, PD98059, was used to inhibit the activation of ERK1/2. The increase in EGR1 after IR was attenuated in the presence of ERK1/2 inhibitor (Figure 4e), confirming the regulation of EGR1 by ERK. In addition, ERK1/2 inhibitor also significantly suppressed RIBE occurrence, displaying the decreased induction of COX-2 protein (Figure 4f) and MN in the bystander cells (Figure 4g). Collectively, a novel signaling pathway (GOLPH3/ERK/EGR1/TNF-α) in irradiated cells was activated in response to irradiation to trigger the occurrence of the RIBE (Figure 5).

## 4. Discussion

In this study, we found that GOLPH3 mediated RIBE induction by regulating the transcription of TNF-α. Loss of GOLPH3 in the irradiated cells significantly inhibited the RIBE in vivo and in vitro, while re-expression of GOLPH3 in the irradiated GOLPH3 knockdown cells restored the RIBE. Mechanistically, a GOLPH3/ERK/EGR1 signaling axis modulates the transcription of TNF-α to regulate the RIBE. These findings enable a better understanding of the molecular mechanism underlying the RIBE. As we know, gene mutations and chromosomal instability caused by the RIBE may result in cancerous transformation and reproductive impairment [5,29,30]; thus, the occurrence of RIBE increases the health risks to normal tissues in the survivors, especially the young, after radiotherapy. The nausea and fatigue after radiotherapy are also thought to be associated with the RIBE [31]. However, the RIBE, unlike the direct effect of radiation, is unable to be prevented by physical protection. Blocking the generation or transmission of the RIBE intercellular signal molecules or inhibiting the activation of the RIBE signal pathways in the bystander cells are considered as effective strategies to prevent the RIBE. From this perspective, vitamin C, vitamin E, carbon monoxide (CO), etc., have been identified to protect the bystander cells or tissues against the attack of RIBE [32,33,34]. In fact, targeting the pathways of the RIBE in irradiated cells is also effective to prevent the RIBE, though sometimes this may affect the killing effect on the tumor due to the decrease in DNA damage or ROS production. In our study, we confirmed that GOLPH3 could be a potential molecular target for modulating the RIBE. Together with our recent identification of GOLPH3 as a radiosensitization target in lung cancer [27], the inhibition of GOLPH3 in radiotherapy appears to kill these “two birds” with one stone, so targeting GOLPH3 may be a promising strategy to achieve therapeutic benefit in radiotherapy.

Several studies have demonstrated that the cytokine profile of tumor cells changes after IR, and multiple soluble factors, known to induce RIBE, are induced and released into the extracellular space [35]. In general, irradiated cells exhibit a diversity of cytokine profiles depending on cell types, radiation doses, radiation types, etc., so the soluble factor involved in RIBE may be different. As we know, TGF-β released from irradiated HeLa or T98G cells acts as a signal molecule to induce the RIBE [36,37], whereas the IL-6 released from the irradiated PC3 cells triggers the RIBE [38]. In our model using the irradiated A549 or H1299, TNF-α (but not TGF-β or IL-6) acts as an intercellular signal molecule to mediate the transduction of the RIBE, while the release of TGF-β and TNF-α after irradiation was still increased. The finding also implies that the soluble factor(s) involved in the RIBE is determined by not only the irradiated cells but also the bystander cells, displaying cell-type specificity. Previously, the involvement of TNF-α in mediating the RIBE has been confirmed in vitro, and the TNF-α-induced activation of COX-2 in the bystander cells is reported to play a key role in RIBE transduction [39,40,41]. This conclusion is also supported by our findings that secreted TNF-α from the irradiated cells elevated the level of the COX-2 protein in the bystander cells.

The transcriptional factor EGR1 has been previously shown to activate the transcription of TNF-α [40]. Here, in agreement with the previous study, the positive correlation between EGR1 and TNF-α was also observed in the irradiated A549 and H1299 cells, supporting the role of EGR1 in the activation of TNF-α. Furthermore, we found an important role of GOLPH3 in the activation of EGR1 and identified ERK1/2 as a link between GOLPH3 and EGR1. In previous studies, GOLPH3 have been reported to be involved in the activation of multiple signaling pathways, such as NF-κB, GSK3β, STAT3 and ERK1/2 [42,43]. However, we only observed the activation of ERK1/2 rather than NF-κB, GSK3β or STAT3 at 1.5 h after irradiation. These results were in line with those of previous studies, that ERK1/2 [44] was activated at 1 h after IR, whereas activation of NF-κB (phosphorylation of IκB-α) [45] and STAT3 [46] was observed at least 3 h after IR, suggesting an important role for ERK in the early response of cells after IR. Inhibition of ERK1/2 activity with its specific inhibitor in the irradiated cells suppressed the activation of ERG1 and blocked the induction of RIBE, further confirming the key role of ERK1/2 in RIBE induction. These findings are also supported by several previous studies, that EGR1 is the target gene of ERK1/2 [47,48]. Importantly, our results showed that GOLPH3 knockdown blocked the activation of ERK1/2 and EGR1 after irradiation and then inhibited the RIBE, indicating that the GOLPH3/ERK/EGR1/TNF-α signal axis regulates the RIBE. In addition, growth factor receptors, such as epidermal growth factor receptor (EGFR) and platelet-derived growth factor receptor (PDGFR), have been identified to be involved in the substantial activation of the ERK induced by oxidative stress [49,50]. In our previous study, we confirmed that GOLPH3 maintains the stability of the EGFR protein. Therefore, we speculate that GOLPH3 may act as a sensor of the oxidative stress induced by IR, and EGFR may be the mediator between GOLPH3 and the activation of ERK1/2. Certainly, further investigations are needed to verify this speculation.

## 5. Conclusions

Our study reveals that GOLPH3 plays a key role in RIBE induction. Furthermore, a novel signal pathway, GOLPH3/ERK/EGR1, was identified to modulate the transcriptional upregulation of TNF-α after IR, which then caused the release of TNF-α from the irradiated cells to induce the RIBE. Our findings provide new insights into understanding the molecular mechanism of the RIBE and suggest that GOLPH3 could be a potential molecular target for the prevention of the RIBE. A novel combination of GOLPH3 inhibitor and radiotherapy offers an exciting therapeutic strategy to overcome the radioresistance of cancer and reduce the risk of secondary cancer caused by the RIBE.

## Data Availability

The data presented in this study are available in article and supplementary material.

## Supplementary Materials

The following supporting information can be downloaded at: https://www.mdpi.com/article/10.3390/antiox11112172/s1. Figure S1: Knockout of GOLPH3 in irradiated A549 cells blocks the induction of RIBE.
