# Supplementary material for: Golgi Phosphoprotein 3 Mediates Radiation-Induced Bystander Effect via ERK/EGR1/TNF-α Signal Axis
Source: Antioxidants (Basel). 2022 Nov 1;11(11):2172. doi: 10.3390/antiox11112172 (PMC9686538; doi:10.3390/antiox11112172)
Supplement: Supplementary file 1 [file antioxidants-11-02172-s001.zip › antioxidants-1936480-supplementary.pdf]

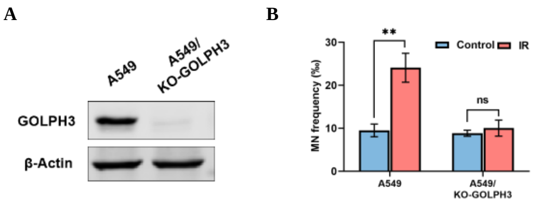

Figure S1. Knockout of GOLPH3 in the irradiated A549 cells blocks the induction of RIBE. **A.** Validation of GOLPH3 knockout with western blotting. **B.** MN yields in the bystander A549 cells after co-culture with the irradiated A549 or A549/GOLPH3-KO cells, respectively. **: $p < 0.01$; ns: not significant.
